# Optimization of Calcined Bone Powder and Silane-Crosslinked Alginate Composites for Enhanced Mechanical Performance as a Cortical Bone Substitute

**DOI:** 10.1007/s10439-025-03924-7

**Published:** 2025-11-28

**Authors:** Shigeo M. Tanaka

**Affiliations:** Faculty of Frontier Engineering, Institute of Science and Engineering, Kanazawa University, Kakumamachi, Kanazawa, Ishikawa 920-1192 Japan

**Keywords:** Bone substitute composite, Calcined bone powder, Silane cross-linked alginate, Nanoscale reinforcement, Naturally derived materials, Mechano-compatibility

## Abstract

**Purpose:**

Developing bone substitute materials that mimic both trabecular and cortical bone remains a major challenge due to the trade-off between bio- and mechano-compatibility, particularly in naturally derived materials. While composites of calcined bone powder and silane-crosslinked alginate exhibit good biocompatibility and mechanical properties resembling those of trabecular bone, their mechanical properties remain insufficient for cortical bone applications.

**Methods:**

This study explores a strategy to address this limitation by optimizing the composite formulation through blending ratio adjustment and nanoparticulation of calcined bone powder. Cylindrical composites (φ 15 mm × h 8 mm) were fabricated by varying the ratios of calcined bone powder (average particle size 246 μm), alginate, and silane cross-linking agent.

**Results:**

Increasing the alginate ratio 10-fold (B/A10-Si) relative to the original formulation (B/A-Si) led to significant increases in elastic modulus, maximum stress, and strain energy which were further improved with the addition of a reduced amount of silane agent (B/A10-Si1/10). Additional enhancement was achieved using nanoparticulated bone powder (average particle size 651 nm), leading to further increases in modulus, strength, and energy by factors of 2.4, 1.7, and 1.4 respectively, compared to B/A10-Si1/10. Overall, the elastic modulus, maximum stress, and strain energy improved 8.4-fold, 18-fold, and 11-fold compared to B/A-Si, approaching values characteristic of cortical bone.

**Conclusion:**

These findings suggest that combining blending optimization with nanoparticulation is a promising strategy to enhance the mechanical performance of naturally derived composites and may expand their applicability to cortical bone replacement.

## Introduction

To ensure post-implantation stability, high-modulus synthetic materials such as stainless steel or titanium alloys are commonly used as cortical bone substitutes [1]. However, the much higher stiffness of these metals can cause stress shielding in adjacent bone tissue [2]. Stress shielding alters the mechanical adaptation of the bone and often causes resorption in the surrounding area [3]. Consequently, artificial joints may loosen gradually, necessitating revision surgeries [4]. To mitigate stress shielding, researchers have attempted to reduce the apparent elastic modulus of Ti alloys by introducing porous structures. Previous studies [5, 6] have successfully optimized porosity to achieve mechanical properties comparable to those of cortical bone. However, a major drawback is the reduced fatigue strength caused by local stress concentrations [7].

In contrast, naturally derived biomaterials are attractive for their excellent biocompatibility, owing to low immunogenicity, non-toxicity, biodegradability, and structural similarity to natural tissues. As a result, natural organic biomaterials such as collagen, alginate, chitosan, hyaluronic acid, cellulose, and silk have been extensively studied for bone-tissue engineering applications [8]. Notably, alginate has often been combined with hydroxyapatite to form composites mimicking trabecular bone [9–12]. Among naturally derived inorganic biomaterials, calcined bone has been widely and clinically used as an inorganic xenogenous bone substitute [13]. While calcination eliminates immunogenicity, it also increases mechanical fragility, making calcined bone unsuitable as a bone substitute. This brittleness can be mitigated by combining it with naturally derived polymers such as fibrin [14]. However, natural biomaterial composites have yet to achieve mechanical properties sufficient for cortical bone replacement.

In our previous study, we developed a novel bone substitute composite (B/A-Si) using natural materials: calcined bone powder and silane-crosslinked alginate [15]. The two materials were combined with silane cross-linking agents to improve mechanical toughness. This combination provided mechanical compatibility with trabecular bone and remained stable in simulated body fluids. Additionally, an in vitro study confirmed that the composite was non-cytotoxic, biocompatible, and exhibited osteoconductivity due to spontaneous apatite formation. These results indicate that this composite is an excellent bone substitute material with mechanical compatibility comparable to trabecular bone, rendering it a promising candidate for applications such as bone fillers. However, the previous composite did not exhibit mechanical properties sufficient for cortical bone replacement, thereby limiting its clinical applicability. Achieving mechanical compatibility with cortical bone is essential not only for load-bearing capacity but also for promoting bone remodeling and ensuring long-term clinical success, which helps reduce the risk of stress shielding and revision surgeries. To address this gap, we aimed to enhance the composite’s mechanical properties by optimizing the blending ratio and nanosizing the calcined bone powder.

## Materials and Methods

### Calcined Bone Powder/Silane Cross-Linked Alginate Composite

**Fig. 1:**
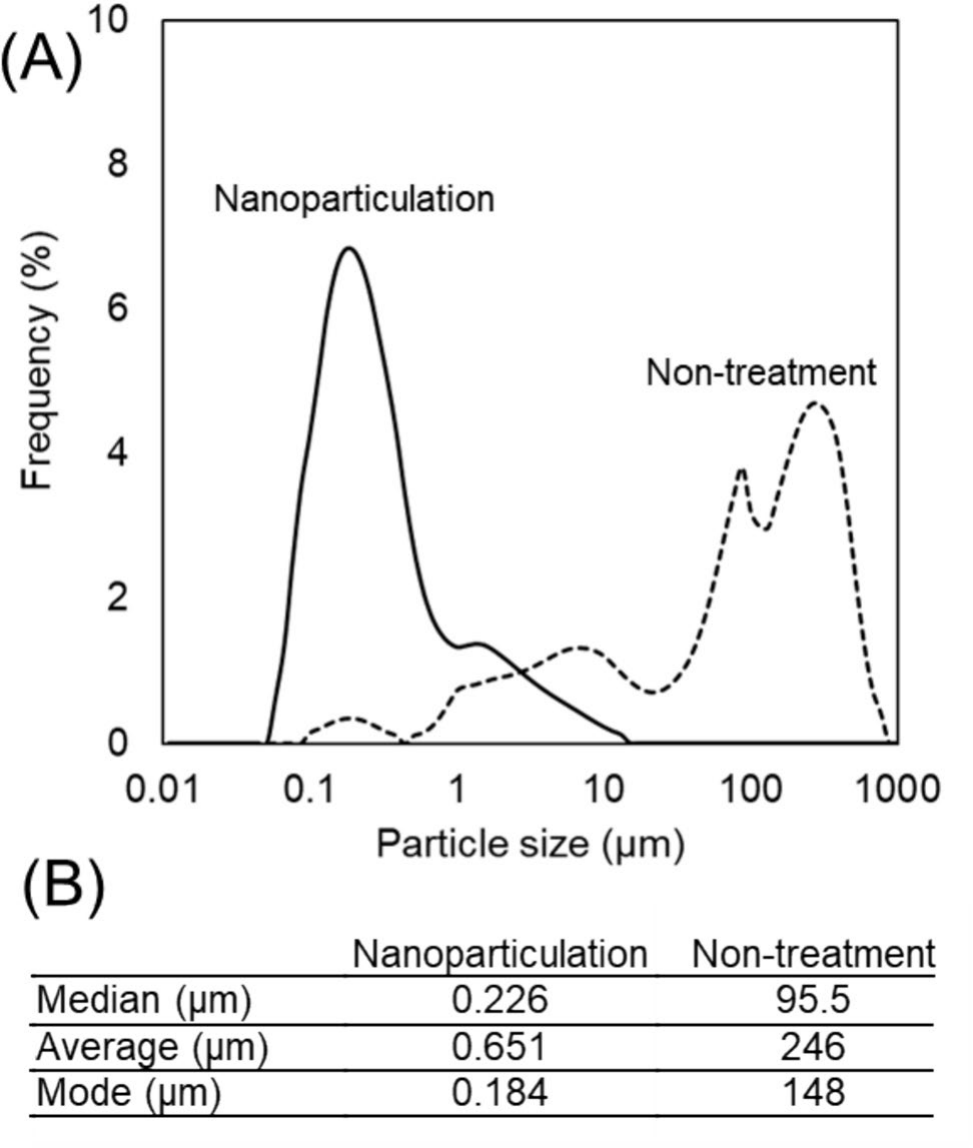
Comparison of the particle size distribution of calcined bone powder before and after nanoparticulation. **A** The nanoparticulation process resulted in a particle size distribution centered around several hundred nanometers. **B** Particle size distribution was measured using a laser scattering particle size distribution analyzer (LA-960, HORIBA, Kyoto, Japan)

The fabrication procedure for the calcined bone powder/silane-cross-linked alginate composite has been described previously [15]. Briefly, bovine femoral trabecular bone blocks, with the bone marrow removed, were calcined in an electric furnace at 600 °C for 22 h. The resulting material was ground and sieved through a 500-μm mesh, producing micro-calcined bone powder with an average particle size of 246 μm (Fig. 1, Non-treatment). This powder was then mixed with alginate modified using silane cross-linking agents [1.5 × 10^−2^ mol/L 3-aminopropyltriethoxysilane, 0.98 × 10^−1^ mol/L 1-ethyl-3-(3-dimethylaminopropyl)-carbodiimide hydrochloride, 1.5 × 10^−2^ mol/L *N*-hydroxysuccinimide] at a mass ratio of 1:1. The mixture was molded into a φ 15 mm × 8 mm mold to produce the original composite samples (B/A-Si). In addition, a cortical bone sample of the same dimensions was prepared from the diaphysis of a bovine femur for comparison. The longitudinal axis of the sample was aligned with the anatomical axis of the bone. The bovine femoral bone used in this study was obtained from a commercially available, Safe Quality Food (SQF)-certified source and handled in accordance with ethical guidelines for animal-derived materials in biomedical research.

### Composites with Different Blending Ratios

**Fig. 2:**
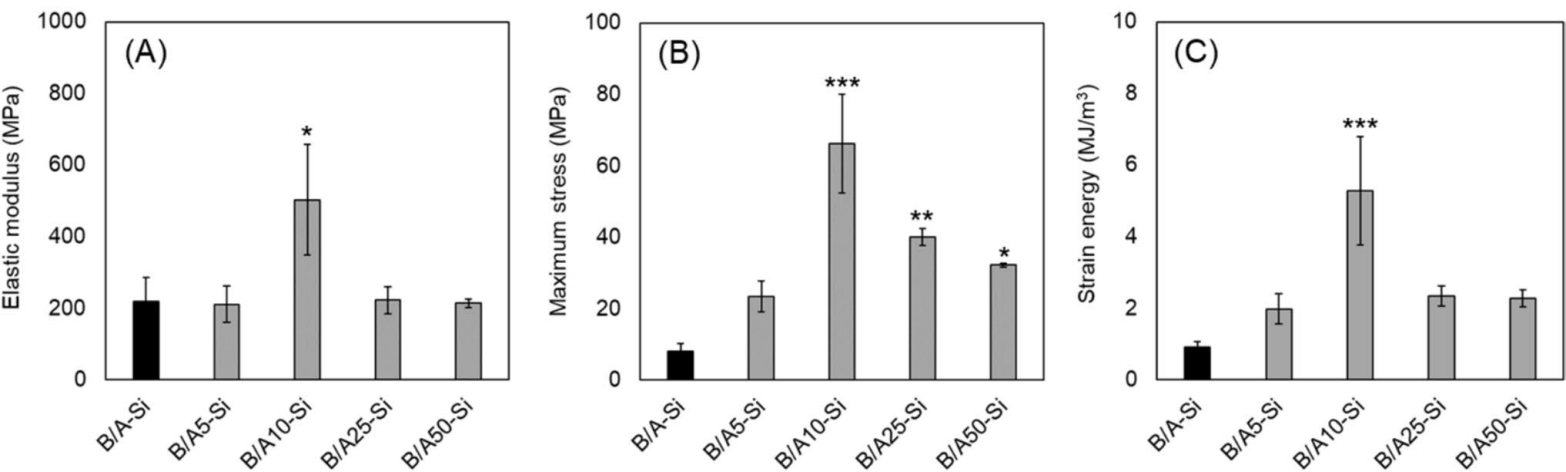
Mechanical properties of the alginate-modulated composites. **A** Elastic modulus, **B** Maximum stress, and **C** Strain energy. B/A10-Si exhibited significantly higher elastic modulus, maximum stress, and strain energy values compared to the original composite, B/A-Si. Symbols: *p < 0.05, **p < 0.01, ***p < 0.001 vs. B/A-Si

To examine the effect of the alginate blending ratio on mechanical properties, composites were prepared with alginate amounts of 5, 10, 25, and 50 times that of the original B/A-Si content. These composites were designated as B/A5-Si, B/A10-Si, B/A25-Si, and B/A50-Si, respectively, and their mechanical properties were compared. Next, to assess the effect of the silane cross-linking agent ratio, composites were fabricated with silane cross-linking agent amounts set at 1/20, 1/10, 1/5, 5, 10, and 20 times those used in the alginate-modified composite exhibiting the highest mechanical properties (B/A10-Si, Fig. 2). These composites were designated as B/A10-Si1/20, B/A10-Si1/10, B/A10-Si1/5, B/A10-Si5, B/A10-Si10, and B/A10-Si20, and their mechanical properties were compared. The aforementioned blending ratios were determined within the range that allowed the composites to solidify in the correct form.

### Composite with Nanoparticulate Calcined Bone Powder

The composite exhibiting the best mechanical properties among different blending ratios of silane crosslinking agents (B/A10-Si1/10, Fig. 4) was selected for further analysis. To evaluate the effect of nanoparticulation on mechanical properties, calcined bone powder was subjected to 20 cycles of wet pulverization using a STAR BURST system (Sugino Machine, Toyama, Japan). In this system, raw materials are injected using water jets at an ultra-high pressure of 245 MPa, causing them to collide and form nanoparticles. Because no pulverizing media is used, contamination is minimized, and a uniform particle size can be achieved. The number of cycles was determined as the minimum value required to reliably achieve nanosized particles. This process reduced the average particle size to 651 nm (Fig. 1).

### Immersion Testing in Simulated Body Fluid

To assess the stability of the mechanical properties of the composite under physiological conditions, the composite with varying alginate and silane cross-linking agent ratios was immersed in simulated body fluid, SBF at approximately 22 °C for 1 h, 1 week, and 2 weeks. Changes in mechanical properties over time were analyzed. All mechanical tests were conducted immediately after removal of the samples from SBF, ensuring that the specimens were evaluated in a fully hydrated state.

### Mechanical Testing

Quasistatic compression tests were performed using a mechanical testing machine (AG-250kNXPlus, Shimadzu, Kyoto, Japan). Compression tests were performed at a crosshead speed of 1 mm/min until a maximum displacement of 3 mm was reached. The elastic modulus was calculated from the slope of the initial linear region of the stress–strain curve, whereas the values of maximum stress and strain energy were taken at a strain of 0.15 because no distinct peak was detected in the compression tests.

### Statistical Analysis

The statistical significance of differences in mechanical properties under varying conditions was analyzed using IBM SPSS Statistics (Version 29.0.2.0, IBM, Armonk, NY, United States). The assumptions of normality and homogeneity of variance were tested prior to conducting statistical analyses to ensure the validity of the analysis of variance (ANOVA). A one-way ANOVA followed by Dunnett’s post hoc test was used to evaluate the effects of blending ratios and immersion durations in SBF, whereas Tukey’s honest significant difference (HSD) post hoc test was employed to assess the effect of nanoparticulation of calcined bone powder on mechanical properties. To assess changes in mechanical properties over immersion time in SBF, a two-way ANOVA was initially conducted to evaluate the interaction between immersion time and composite type. As a significant interaction was detected between these factors, one-way ANOVAs were subsequently performed for composite type at each immersion time. The sample size was three for all conditions. Statistical significance was set at p < 0.05.

## Results

### Mechanical Properties of Composites with Varying Blending Ratios of Alginate

**Fig. 3:**
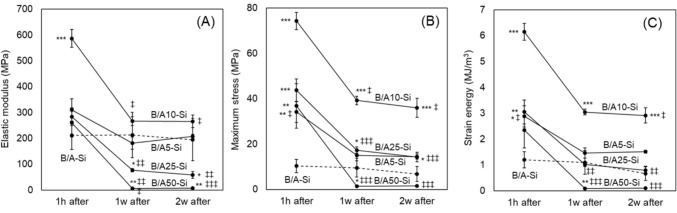
Changes in the mechanical properties of alginate-modulated composites over 2 weeks of immersion in SBF. **A** Elastic modulus, **B** Maximum stress, and **C** Strain energy. The values significantly decreased during the first week, in contrast to those of the original composite (B/A-Si), and then stabilized in the second week. Symbols: *p < 0.05, **p < 0.01, ***p < 0.001 vs. B/A-Si; ^‡^p < 0.05, ^‡‡^p < 0.01, ^‡‡‡^p < 0.001 vs. pre-immersion state (Fig. 2)

Compared to the original B/A-Si composite, the elastic modulus of B/A10-Si was significantly higher by approximately 2.3-fold, whereas the elastic moduli of the other composites remained comparable to that of B/A-Si (Fig. 2A). As the alginate blending ratio increased, the maximum stress also increased. The B/A10-Si, B/A25-Si, and B/A50-Si composites exhibited significantly higher maximum stress values compared to B/A-Si (Fig. 2B), with B/A10-Si showing the highest maximum stress, approximately 8.2-fold higher than that of B/A-Si. In terms of strain energy, all alginate-blended composites exhibited higher values than B/A-Si (Fig. 2C). Among them, B/A10-Si showed the highest strain energy, approximately 5.8-fold greater than that of B/A-Si. Composites with higher alginate content exhibited significant mechanical degradation following immersion (Fig. 3A, B). After 1 week in SBF, the elastic modulus decreased by 14–97%, the maximum stress declined by 36–95%, and the strain energy declined by 26–96% from their pre-immersion values (Fig. 2), with greater reductions observed at higher alginate blending ratios. However, during the second week of SBF immersion, the mechanical properties stabilized, and no further significant decrease was observed. Throughout the immersion period, only B/A10-Si consistently maintained mechanical properties equal to or greater than those of the original composite, B/A-Si.

### Mechanical Properties of Composites with Varying Blending Ratios of Silane Cross-Linking Agents

**Fig. 4:**
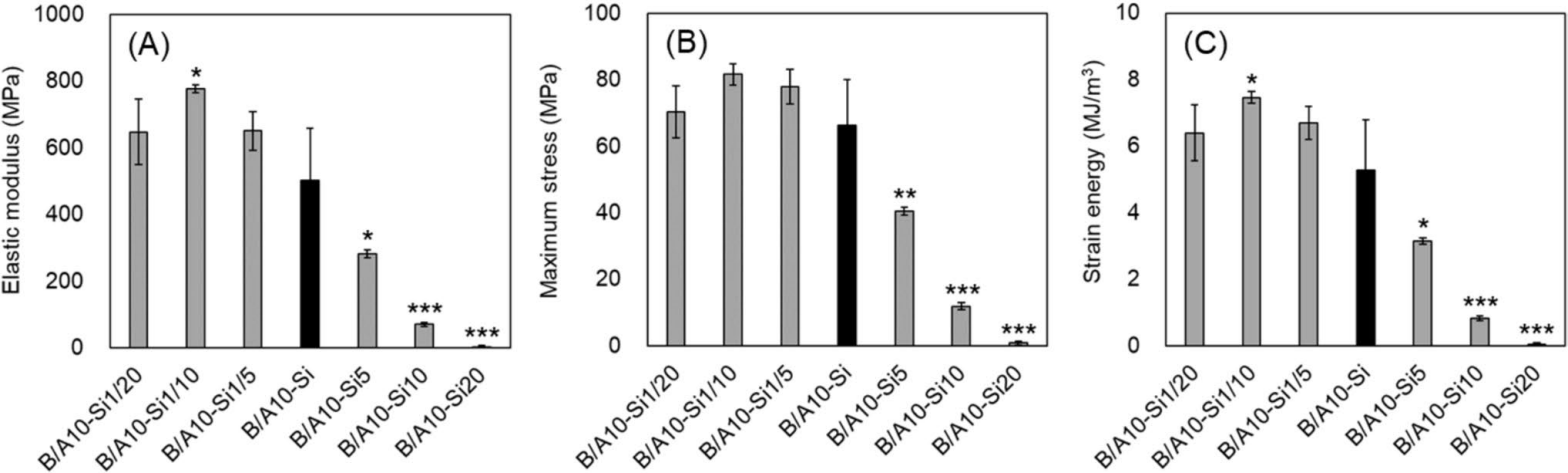
Mechanical properties of silane-modulated composites. **A** Elastic modulus, **B** Maximum stress, and **C** Strain energy. B/A10-Si1/10 exhibited the highest values for all three mechanical properties (elastic modulus, maximum stress, and strain energy); however, the maximum stress did not differ significantly from that of the reference composite, B/A10-Si. Symbols: *p < 0.05, **p < 0.01, ***p < 0.001 vs. B/A10-Si

**Fig. 5:**
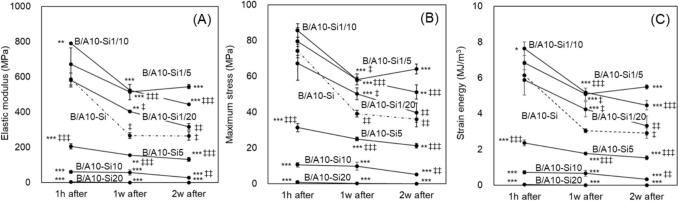
Changes in the mechanical properties of silane-modulated composites over 2 weeks of immersion in SBF. **A** Elastic modulus, **B** Maximum stress, and **C** Strain energy. Composites with lower ratios of silane cross-linking agents maintained higher values of mechanical properties throughout the immersion period, whereas those with higher ratios remained relatively stable but exhibited lower values. Symbols: *p < 0.05, **p < 0.01, ***p < 0.001 vs. B/A10-Si; ^‡^p < 0.05, ^‡‡^p < 0.01, ^‡‡‡^p < 0.001 vs. pre-immersion state (Fig. 4)

When using B/A10-Si as a reference, composites with reduced silane cross-linking agent content exhibited increased elastic modulus and maximum stress, whereas those with high silane content showed significant reductions in these properties (Fig. 4A–C). Among them, B/A10-Si1/10 demonstrated the highest values for both mechanical properties, with elastic modulus and strain energy significantly higher than that of B/A10-Si. After 1 week of SBF immersion, the elastic modulus declined by 17–83%, the maximum stress was reduced by 18–85%, and the strain energy decreased by 19–84% in all composites from their pre-immersion values (Fig. 4), excluding B/A10-Si, which was treated as a reference control (Fig. 5A–C). Composites with lower silane cross-linking agent ratios (B/A10-Si1/5, B/A10-Si1/10, and B/A10-Si1/20) exhibited relatively more stable mechanical properties compared with B/A10-Si throughout the immersion period, while maintaining values equal to or greater than those of B/A10-Si.

### Mechanical Properties of Composites with Nanoparticulation

**Fig. 6:**
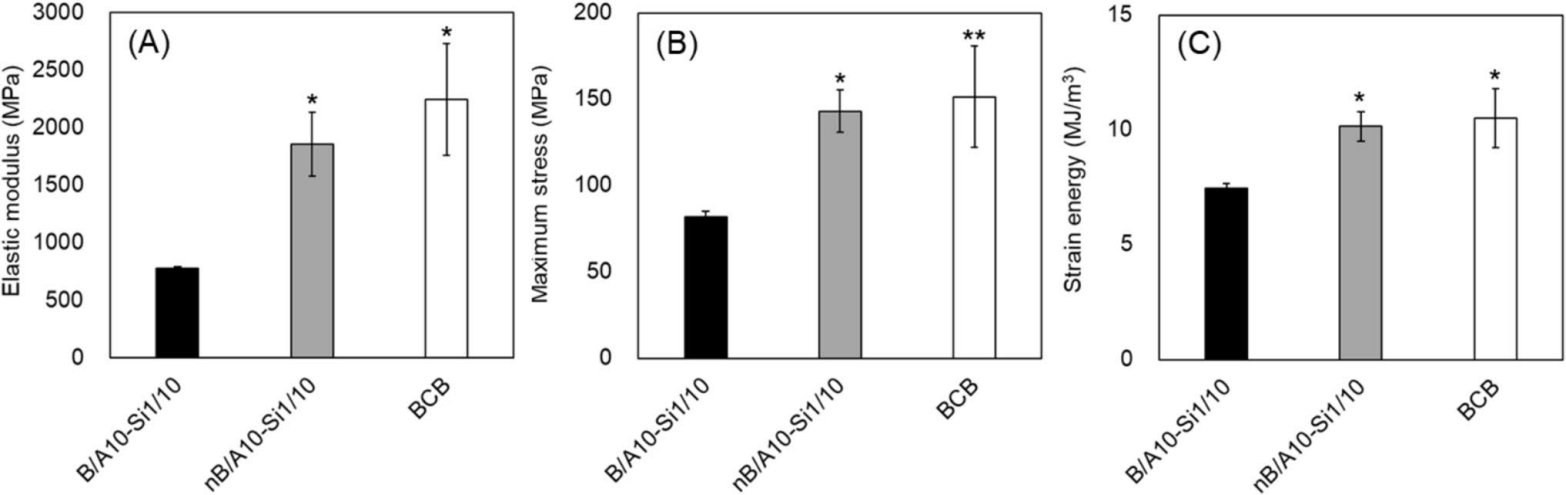
Mechanical properties of the nanoparticulate composite (nB/A10-Si1/10). **A** Elastic modulus, **B** Maximum stress, and **C** Strain energy. Nanoparticulation improved mechanical properties to BCB-comparable levels. Symbol: *p < 0.05, **p < 0.01 vs. B/A10-Si1/10

**Fig. 7:**
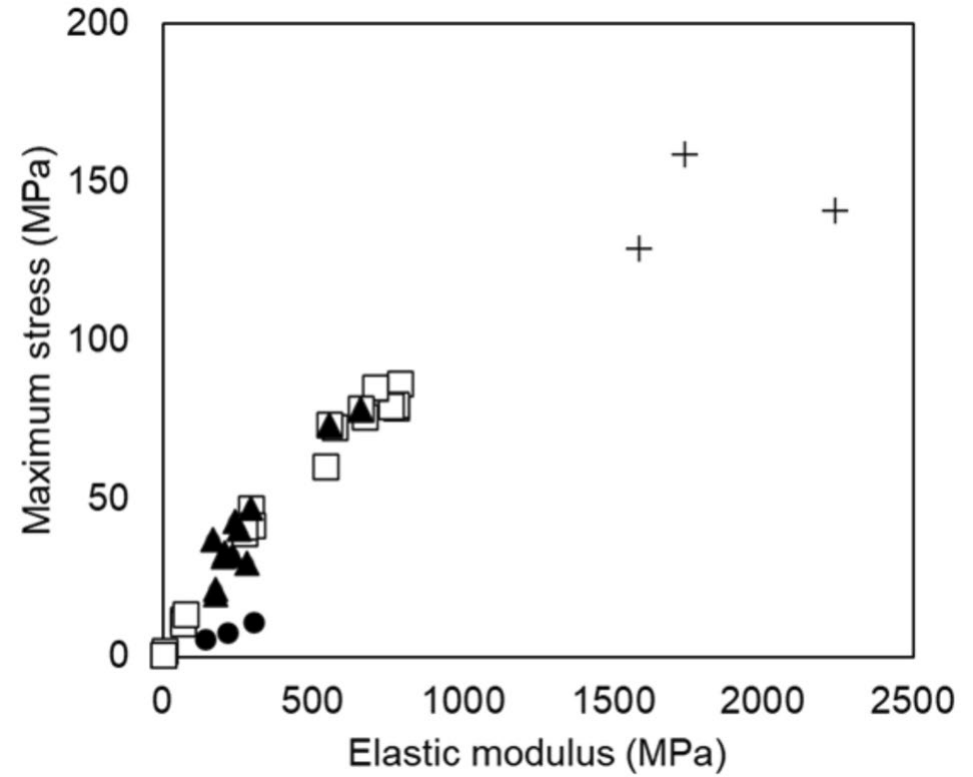
Relationship between the elastic modulus and maximum stress of composites fabricated using calcined bone powder and silane cross-linked alginate. The mechanical properties can be broadly adjusted by modifying the blending ratio of alginate (*filled triangles*) and silane cross-linking agents (*open squares*) and can be further enhanced through the nanoparticulation of calcined bone powder (plus), relative to the original composite (filled circles)

The nanoparticulation of calcined bone powder improved the mechanical properties of the composite (Fig. 6A–C). Compared to the reference composite, B/A10-Si1/10, the nanoparticulated composite (nB/A10-Si1/10) exhibited a 2.4-fold increase in the elastic modulus and a substantial 1.7-fold increase in maximum stress, and a 1.4-fold increase in strain energy, achieving values comparable to those of bovine cortical bone (BCB). Figure 7 illustrates the relationship between the elastic modulus and maximum stress for composites with varying alginate and silane cross-linking agent ratios, as well as those incorporating nanoparticulated calcined bone powder. These properties generally exhibited a proportional relationship, indicating that the mechanical characteristics of the composites could be broadly adjusted through compositional modifications and nanoparticulation. By optimizing the blending ratios and incorporating nanoparticulation, both mechanical properties were significantly enhanced, with the elastic modulus increasing by approximately fourfold and the maximum stress increasing by approximately 16-fold compared to those of the original B/A-Si composite.

## Discussion

In this study, we aimed to enhance the mechanical properties of calcined bone powder and silane-cross-linked alginate composites to achieve compatibility with cortical bone. The scope of this work was limited to mechanical optimization, and biological evaluations such as cytocompatibility and osteogenic potential were not included, as they were partly addressed in our previous study [15]. To this end, we optimized both the material and cross-linker blending ratios, and applied nanoparticulation to the calcined bone powder. The composite with a 10-fold alginate-to-bone powder weight ratio (B/A10-Si) exhibited the highest elastic modulus, maximum stress, and strain energy among all formulations. Further improvements were observed by reducing the cross-linking agent to one-tenth (B/A10-Si1/10). Incorporating nanoparticulated calcined bone powder (nB/A10-Si1/10) further enhanced the mechanical performance. The mechanical properties of the composites were influenced by the alginate proportion, reaching a peak at a specific blending ratio (Fig. 2). When the alginate content was low, the composite became brittle and prone to cracking, leading to mechanical degradation. Conversely, when the alginate content was excessively high, excess alginate did not effectively interact with the calcined bone powder, increasing ductility and reducing mechanical strength. Similarly, experimental results indicate that the amount of silane cross-linking agent influences mechanical properties, implying the existence of an optimal concentration (Fig. 4).

Nanoparticulation of calcined bone powder improved composite mechanical properties to levels comparable to those of cortical bone (Fig. 6). This improvement is likely attributable to the increased surface area, which promotes stronger interactions with alginate chains and suppresses crack initiation and propagation. Nanoparticulation also likely contributed to the improvement in elastic modulus by increasing packing density. Similar findings have been reported for hydroxyapatite–chitosan–starch composites, where nanopowder incorporation improved tensile modulus compared to micropowder-based composites [16]. Similarly, bone matrix is a composite of collagen fibers and hydroxyapatite. During bone formation, osteoblast-derived matrix vesicles deposit in the hole zones between collagen fibers, promoting composite formation through crystal growth along the fiber direction [17, 18]. The high toughness of bone arises from nanoscale bonding between collagen and hydroxyapatite. Thus, enhancing nanoscale bonding between nanocalcined bone powder and alginate is critical for achieving cortical bone-level performance.

Immersion in simulated body fluid caused a significant decline in the mechanical properties of composites with higher alginate content during the first week, after which they stabilized (Fig. 3). The composite is cross-linked both covalently by silane and ionically by calcium ions. The observed reduction in mechanical properties is attributed to the replacement of calcium ions with sodium ions in SBF, which disrupts cross-links and induces swelling [19]. A higher alginate content likely exacerbated this effect. In contrast, reducing the silane cross-linking agent mitigated the mechanical degradation of the composite in SBF (Fig. 5). This effect is likely due to the reduction of unreacted cross-linking agents, thereby stabilizing mechanical properties in fluid environments. Alginate swelling can be suppressed by increased calcium ion concentration [20], cross-linking with barium ions [21], or low pH [22]. Additionally, the nanoparticulation of powders reduces swelling [16]. Future studies should focus on suppressing swelling, stabilizing mechanical properties in body fluids, and enhancing biocompatibility and biointegration in vivo.

This study has several limitations. First, only quasistatic compression tests were performed, and fatigue testing was not included. Because cortical bone in vivo is constantly subjected to cyclic loading, fatigue resistance is a critical property to evaluate the long-term performance and clinical relevance of bone substitute materials. The absence of fatigue testing thus restricts the scope of the present findings. Second, immersion stability was assessed only in simulated body fluid at room temperature for up to 2 weeks, which does not fully reproduce physiological conditions. I consider the room-temperature results to at least reflect the early response that would also occur under physiological temperature. Further investigations, including immersion experiments at 37 °C, fatigue testing under physiologically relevant conditions, and extended long-term immersion studies, will be necessary to validate the clinical potential of the optimized composite formulations. Third, each condition was tested with only three specimens, which restricts the statistical robustness of the optimization. Future studies with larger sample sizes, effect size estimation, and power analysis will be necessary to strengthen the reliability of the findings.

This study demonstrated that optimized calcined bone powder/silane-cross-linked alginate composites—especially the nB/A10-Si1/10 formulation—achieve mechanical properties suitable for load-bearing cortical bone applications. These results offer promising insight for developing next-generation bone grafts derived from natural materials, including scaffolds, bone fillers, and joint prostheses. Furthermore, the ability to fine-tune mechanical properties via blending ratios and nanoparticulation highlights the adaptability of these composites for diverse clinical uses such as orthopedic repair, bone regeneration, and maxillofacial reconstruction.

## Data Availability

The datasets generated and analyzed during the current study are available from the corresponding author on reasonable request. Not applicable.
